# The British Medical Association and the Elections

**Published:** 1906-01-20

**Authors:** 


The Hospital.

A Journal of

The Medical Sciences and Hospital Administration.

Vol. XXXIX.—No. 1,008. SATURDAY, JANUARY 20, 1906.

### The British Medical Association and the Elections.

We lately had occasion to point out, in an article on the approaching election, that it would be perfectly legitimate, at such a time, for the members of all professions or callings to bethink them of their own special requirements; and, so long as these were kept in due subjection to larger national interests, to seek to bring them to the notice of candidates. We added that the reservation was necessary, because nothing could be more opposed to genuine patriotism, or to any real desire for the welfare of the community as a whole, than the endeavours, most frequently made at by-elections, to render the result dependent upon some side issue. We could hardly have anticipated, when these words were written, that the precise course which we felt it necessary to condemn would be recommended, with quite characteristic obtuseness, by the wire-pullers of that curious body, the British Medical Association, or that it would actually be pursued, in certain districts and constituencies, by local committees reflecting the wisdom of the centre. The Supplement to the *British Medical Journal*, for January 6, contains memoranda and questions on Parliamentary subjects affecting the medical profession, submitted for the consideration of Parliamentary candidates, and drawn up " under the authority " (authority is good) of the " Medico-Political Committee " of the Association. The " questions " are twenty-three in number, embracing a great variety of debatable points on proposed changes in medical legislation, on the registration of deaths, on the qualifications and tenure of office of sanitary officials, on re-vaccination, on the reorganisation of the Local Government Board, on midwives, on incipient insanity (with an *excursus* on what the authors are pleased to call " mental disease "), on habitual inebriety and drug-taking, on Poor-law medical officers in Scotland and Ireland, on the medical inspection of school children, on public vaccinators, and on pensions for " asylum workers." It would seem, moreover, that the questions are not really intended for the " consideration " of candidates, but rather as declarations of policy for their unconditional acceptance; for we are told that in some cases " Divisions are making definite recommendations as to the candidates to be supported, basing the recommendations on the replies received to the questions."

Such a document as the foregoing might be thought, at first sight, almost to exhaust the possibilities of human folly; but it has been surpassed and thrown into the shade by another which has followed it in relation to the parliamentary candidates for certain constituencies, and which, there is only too much reason to fear, has been issued, with the necessary local variations, in many parts of England. This second document to which we refer relates, among other constituencies, to that of Mr. Burns, the President of the Local Government Board, who not altogether unreasonably, in view of the restrictions placed upon him by his official position, has omitted to reply to the questions in either the affirmative or the negative. The gross impropriety of attempting to obtain from a minister new to office a series of pledges which would bind not only himself but his department, and to do this before he has had any possibility of consulting either his colleagues in the Government or the permanent officials of his staff on any of the points at issue, would seem to be manifest even to the very humblest capacity; but this, in relation to Mr. Burns, is what the " Division " of the Association has done. Mr. Burns has left the " Division " severely alone, sending no reply to their letters or telegrams; and the " Committee " of the " Division," ten gentlemen of whom we never heard before, have placed on record their " strong disapprobation " of his conduct, and have issued a recommendation that all the medical practitioners in the constituency should vote for his opponent, and should use their influence on his behalf. This is recommended, not on the ground that the opponent in question is a member of the Unionist party and is favourable to fiscal reform, but on the ground that he has swallowed the whole of the twenty-three questions and has replied in the affirmative to all of them. In a neighbouring constituency, all replies are in the affirmative from both candidates, and there the Committee of the Division magnanimously leave the medical electors at liberty to choose for themselves between the opponents. In another the Committee is in a sore dilemma. One of the candidates is not prepared to support compulsory re-vaccination, while another will not commit himself to the proposed legislation concerning habitual inebriety without more knowledge than he now possesses. This, no doubt, is very shocking, but still the "Committee" has mercy upon human weakness, and inclines towards the gentleman who "wants to know" rather than towards him who ventures upon an opinion of his own which is not in accordance with theirs.

We cannot but regret, very deeply, that an organisation which professes to be representative of the medical profession in this country, and which, to many of the uninitiated, may appear to be really so, should make a public appearance of this kind in such a crisis as the present. Nothing is more certain than that it is the duty of medical men to use all fitting opportunities of advocating, by all proper means, the institution of reforms which would either improve the position of the profession or afford increased security against disease to the community at large. Nothing is more certain than that it is disloyal to the Empire to give precedence to the pensions of "asylum workers" over questions on which the whole future of the Empire may depend. And, with regard to "mental disease," they might remember the answer said to have been given by a Lord Chancellor to an Archbishop who sought for facilities for the passing of a Bill relating to clerics suffering under "diseases of the mind." "There are two difficulties," replied the Lord Chancellor. "In the first place, there are no diseases of the mind. In the next place, your Grace is the only member of your sacred calling that I have met with who possesses one." *Mutato nomine*, the Committee of the Division may usefully ponder upon the reply.

